# Immune response to SARS-CoV-2 variants after immunization with different vaccines in Mexico

**DOI:** 10.1017/S0950268824000219

**Published:** 2024-02-05

**Authors:** Erika Garay, Sean P. J. Whelan, Rebecca M. DuBois, Sara M. O’Rourke, Angel Eduardo Salgado-Escobar, José Esteban Muñoz-Medina, Carlos F. Arias, Susana López

**Affiliations:** 1 Departamento de Genética del Desarrollo y Fisiología Molecular, Instituto de Biotecnología, Universidad Nacional Autónoma de México, Cuernavaca, Morelos, Mexico; 2 Department of Molecular Microbiology, Washington University in St. Louis, Saint Louis, United States; 3 Department of Microbiology, Harvard Medical School, Boston, United States; 4 Department of Biomolecular Engineering, University of California, Santa Cruz, United States; 5 Coordinación de Calidad de Insumos y Laboratorios Especializados, Instituto Mexicano del Seguro Social, Mexico City, Mexico

**Keywords:** COVID-19 vaccines, COVID-19, hybrid immunity, Mexico, SARS-CoV-2 variants, seroconversion

## Abstract

There is limited information on the antibody responses against severe acute respiratory syndrome coronavirus 2 (SARS-CoV-2) in subjects from developing countries with populations having a high incidence of co-morbidities. Here, we analysed the immunogenicity of homologous schemes using the ChAdOx1-S, Sputnik V, or BNT162b2 vaccines and the effect of a booster dose with ChAdOx1-S in middle-aged adults who were seropositive or seronegative to the SARS-CoV-2 spike protein before vaccination. The study was conducted post-vaccination with a follow-up of 4 months for antibody titre using enzyme-linked immunosorbent assay (ELISA) and pseudovirus (PV) neutralization assays (PNAs). All three vaccines elicited a superior IgG anti-receptor-binding domain (RBD) and neutralization response against the Alpha and Delta variants when administered to individuals with a previous infection by SARS-CoV-2. The booster dose spiked the neutralization activity among individuals with and without a prior SARS-CoV-2 infection. The ChAdOx1-S vaccine induced weaker antibody responses in infection-naive subjects. A follow-up of 4 months post-vaccination showed a drop in antibody titre, with about 20% of the infection-naive and 100% of SARS-CoV-2 pre-exposed participants with detectable neutralization capacity against Alpha pseudovirus (Alpha-PV) and Delta PV (Delta-PV). Our observations support the use of different vaccines in a country with high seroprevalence at the vaccination time.

## Introduction

The application of different vaccines against severe acute respiratory syndrome coronavirus 2 (SARS-CoV-2) in homologous and heterologous schemes has proved to be a successful strategy for reducing viral transmission and the progression to severe COVID-19 [1–3]. Limited studies have focused on longitudinal immune monitoring in individuals from developing countries, in which obesity, diabetes, and hypertension are prevalent co-morbidities. For these real-world scenarios, the question of mounting an effective and durable immune response against SARS-CoV-2 is still open.

The vaccination campaign in Mexico began on 15 February 2021 for health workers and all citizens over 60 years of age [4]. Initially, five different vaccines were administered: BNT162b2, Pfizer/BioNTech; ChadOx1-S, AstraZeneca; Convidecia, CanSino Biologicals; Sputnik V, Gamaleya Research Institute; and CoronaVac, Sinovac. By the end of June 2021, when the Delta variant was collapsing the health services in other countries, it had just started to circulate in Mexico, and a total of 50 million doses of several different vaccines had been administered in the country, sufficient to cover most of the 50- to 60-year-old group [5].

Studies in COVID-19-recovered subjects have found a reduction in the virus-neutralizing antibody (NtAb) levels over time [6, 7], with a remarkable increase in titres after the administration of the SARS-CoV-2 vaccine [8–10]. It was shown that pre-existing memory B cells participated increase [11] as well as the time interval that elapsed between primary and secondary antigenic exposures [12]. This combined immune response confers what has been termed hybrid immunity since it derives from previous SARS-CoV-2 infections and an artificial immunization by vaccination.

The spike (S) glycoprotein protein of SARS-CoV-2 mediates virus entry into the host cell [13], and the receptor-binding domain (RBD) of this protein is the most relevant antigen eliciting NtAb against the virus [14]. The NtAb response prevents SARS-CoV-2 infection in cultured cells [15], and it is considered one of the best correlates of protection against the virus in animal models [16, 17], together with other antibody-mediated functions like Fc-mediated effector responses [18].

In clinical trials, BNT162b2, Sputnik V, and ChAdOx1-S vaccines showed great efficacy in preventing hospitalization of patients infected with the parental Wuhan strain of the virus [19–21]. After the appearance of SARS-CoV-2 variants, reduced virus-neutralizing activity of sera obtained from people vaccinated with COVID-19 vaccines was observed [22–24], although a vaccine booster dose improved the neutralization activity against the variants [25–27]. To face the immune evasion of Omicron, a homologous or heterologous booster dose with BNT162b2 or ChAdOx1-S was authorized in Mexico for those who already had a full primary vaccination scheme [28].

Here, we analysed, in a longitudinal study, the serum IgG antibody response against the RBD of the spike protein of SARS-CoV-2 induced by a two-dose scheme of either BNT162b2 (20 participants), Sputnik V (21 participants), or ChAdOx1-S (22 participants) vaccines, followed by a booster dose with the ChAdOx1-S vaccine. The participants in this study were either seropositive or seronegative for anti-S antibodies before vaccination. In addition, we assessed the capacity of the SARS-CoV-2-specific antibody response to neutralize the infectivity of the Wuhan, Alpha, and Delta variants in an in vitro assay using SARS-CoV-2 S-protein-bearing vesicular stomatitis virus (VSV) pseudoviruses (PVs). This study provides general insights into the immunogenicity induced by three different vaccine regimens used in Mexico in participants with different backgrounds of prior exposure to SARS-CoV-2 and individual co-morbidities, which has potential implications for future public health policies, specifically for identifying the target subjects for applying a booster dose.

## Materials and methods

### Study design and participants

**Figure 1.**
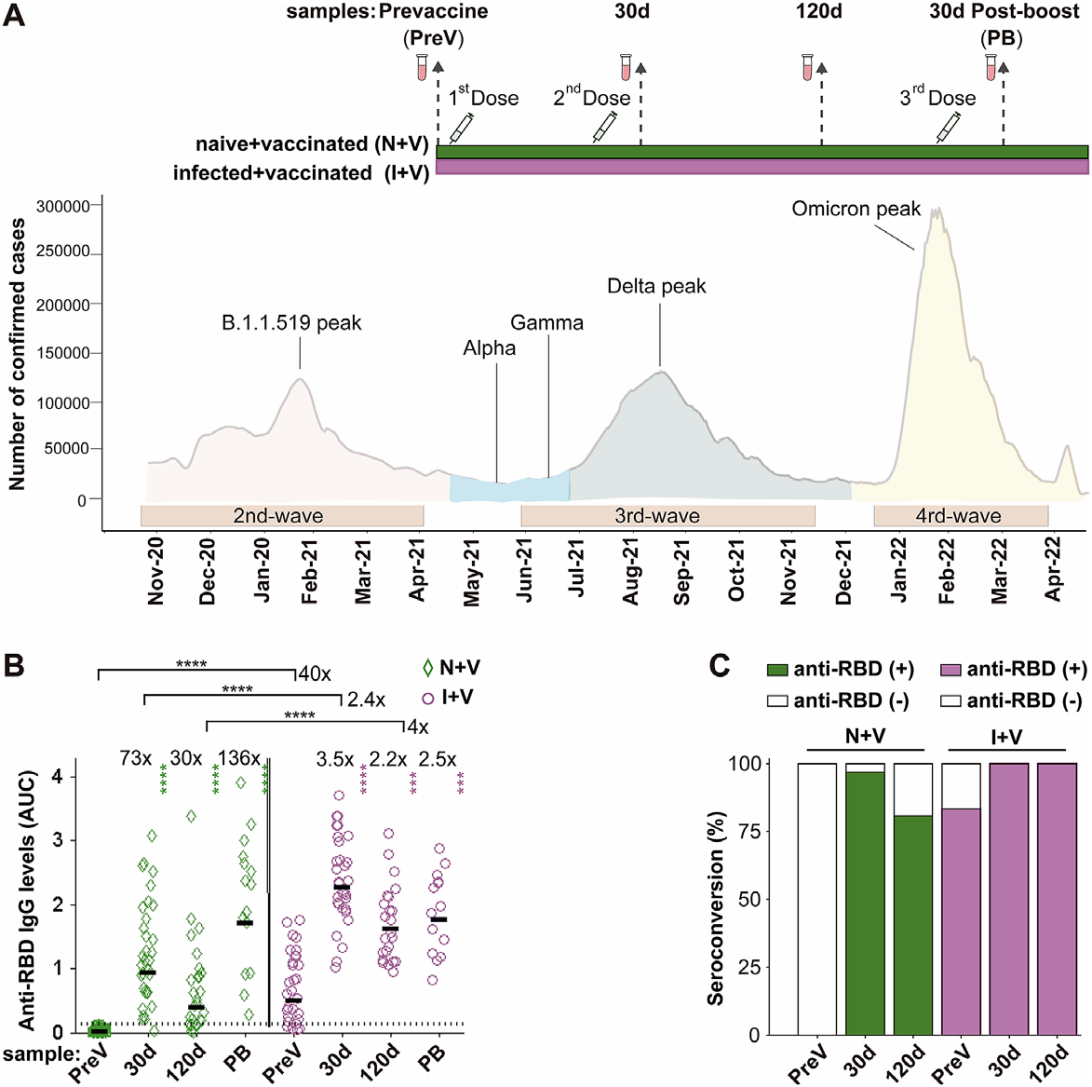
IgG antibodies against S-RBD in the N + V and I + V groups. (a) The scheme of the study design used to investigate the antibody response after vaccination with BNT162b2, ChAdOx1, or Sputnik V and its temporal relationship with the epidemiological waves of infection of SARS-CoV-2 in Mexico. Two groups of participants, who had been infected (I + V) or not (N + V) with SARS-CoV-2 before the initial vaccine dose, were studied. Syringes indicate the time of application of a dose of vaccine, and vials show the serum collection schedule. Sera were collected before the application of the first vaccine (pre-vaccination; PreV), at 30 (30d) and 120 (120d) days after the second dose, and 30 days after the third booster dose (PB). (b) Levels of anti-RBD IgG in the sera collected at time PreV, 30d, and 120d after the second dose, and 30 days post-boost (PB). Bars represent the geometric mean of the areas under the curve (AUC) values (for details, see the Materials and Methods section) of IgG antibodies to RBD. The punctuated line corresponds to the cut-off value for the detection of antibodies against RBD in the ELISA employed. AUC RBD IgG values in individual serum samples for the infection-naive and SARS-CoV-2 pre-exposure groups are shown as green diamonds and purple circles, respectively. For both groups, a two-tailed Kruskal–Wallis test and a Dunn post hoc test were performed on AUC antibody data to test statistical differences; Wilcoxon’s test was used to compare differences in paired samples, ****p < 0.0001,*p < 0.05, **p < 0.01, ***p < 0.001, ****p < 0.0001. (c) Relative IgG-RBD percent positivity at different times in the N + V and I + V groups, as determined by the in-house ELISA. The seropositivity cut-off value was established to be AUC ≥ 0.13 (GM + 4STD).

Sixty-three individuals were recruited for the study at vaccination centres of the Mexican Institute of Social Security (IMSS) in the State of Mexico (EdoMex), a state adjacent to Mexico City, from May to August 2021. The inclusion criteria were subjects aged 50 to 60 years who arrived at the vaccination site; all were included independently of a history of diabetes, hypertension, cardiovascular disease (CVD), autoimmune disease (AD), and/or chronic respiratory disease (RD). Exclusion criteria were the inability to obtain informed consent, as well as the occurrence of a documented secondary SARS-CoV-2 infection after vaccination. For each participant, demographics, co-morbidities, and time from previous COVID-19 infection by clinical symptomatology or a positive nucleic acid or antigen test were documented. Twenty to twenty-two participants received two doses of either ChAdOx1-S, AstraZeneca; Sputnik V, Gamaleya; or BNT162b2, Pfizer-BioNTech vaccines, following the manufacturer-recommended time interval (for details, see Supplementary Table 1). Then, 5.6 months after the second dose, half of the volunteers opted for a booster dose with the ChAdOx1-S vaccine. Blood samples were collected four times: on the day of vaccination (PreV), 30 and 120 days after the second dose, and 30 days after the booster dose (PB) (Figure 1a). The samples were centrifuged at 3000 g for 10 min; serum was separated, aliquoted, and stored at −20^°^C until analysed. The reactivity against the spike (S) glycoprotein of SARS-CoV-2 (200 μl of serum, LIAISON SARS-CoV-2 S1/S2 IgG test) of the PreV samples was used to classify the participants in the I + V group (pre-infected, then vaccinated, n = 31) or in the N + V group (infection-naive, then vaccinated, n = 32).

### Recombinant SARS-CoV-2 spike RBD protein

Recombinant SARS-CoV-2 spike RBD (S-RBD) protein was generated as described previously [29]. Briefly, a pCAGGS expression plasmid encoding the signal peptide (residues 1–14) and RBD (residues 319–541) of the SARS-CoV-2 spike (GenBank: MN908947.3) and fused to a C-terminal 6X His-tag was transfected into suspension-adapted (Chinese Ovary Cells, lineage S) CHO-S cells. On day 8 post-transfection, cells were centrifuged, and the media were 0.22-μm-filtered, diluted with Buffer A (300 mM NaCl, 50 mM NaH_2_PO_4_, 20 mM imidazole [pH 7.4]), and loaded onto a HisTrap column. The column was washed, and S-RBD was eluted with a gradient to Buffer B (300 mM NaCl, 50 mM NaH_2_PO_4_, 225 mM imidazole [pH 7.4]). S-RBD was further purified by size-exclusion chromatography on a Superdex 200 column in phosphate-buffered saline (PBS), and the fractions containing pure monomeric S-RBD were pooled and concentrated to 1.03 mg/ml.

### Anti-RBD IgG detection and analysis

The presence of antibodies to S-RBD was confirmed using an in-house anti-SARS-CoV-2 enzyme-linked immunosorbent assay (ELISA) as previously reported [30]. Briefly, 96-well plates were coated with 50 μl of a 2 μg/ml solution of viral antigen (SARS-CoV-2 RBD protein) in PBS. Plates were incubated at 4 °C overnight. Coated plates were washed 3x with PBS 0.1% Tween-20 (PBST), and wells were blocked with 200 μl of 3% nonfat milk in PBST for 2 h at room temperature (RT). Assay controls and four-step serial dilutions (from 1:150 to 1:9600) of serum-inactivated samples were prepared in 1% non-fat milk prepared in PBST. Assay controls included one non-SARS-CoV-2-reactive human serum sample and a human serum sample reactive to the RBD of SARS-CoV-2 (diluted 1:150). Next, 100 μl of each serum dilution was added to the ELISA plates after removing the blocking solution. After a two-hour incubation at room temperature, the plates are washed with PBST, and a 1:8000 dilution of anti-human IgG (gamma chain-specific; Aviva Systems Biology, Cat. Num. OARA04964) horseradish peroxidase (HRP)-labelled secondary antibody diluted in 1% non-fat milk in PBST was added to each well for 1 h. After washing with PBST, 100 μl of HRP substrate (TMB,3,3′,5,5′-tetramethylbenzidine, Thermo Fisher, Cat. Num. 34021) solution was added for 25 min at 37 °C, and the enzymatic reaction was stopped by the addition of 50 μl per well of 3 M hydrochloric acid. The plates were read in a FLUOstar Omega Microplate Reader (BMG Labtech) at 490 nm.

The values obtained from the serum samples were corrected by subtracting their background value, which corresponded to the optical density obtained from the wells with no antigen (only serum). The corrected values that were negative were set to 0. The corrected results were fitted into a 4-parameter logistic regression curve (4PL), and the area of the resulting curve (area under the curve, AUC) was determined using a trapezoidal numerical integration function (*trapz*) in MATLAB software. The AUC method can be used when the experiment lacks a standard curve, and it integrates the absorbance values obtained from four dilutions in one single data per serum, providing more accuracy in the estimation [31]. Samples that exceeded the AUC cut-off value of 0.13 (the geometric mean of the optical density (OD) values of the negative sera plus four standard deviation values) were assigned as presumptive positive.

### Pseudovirus (PV) production

The eGFP-VSV PVs bearing the SARS-CoV-2 S protein of Wuhan wild type (WT), Alpha, or Delta variants were constructed in the laboratory of S. Whelan [32]. Viral stocks were amplified at a low multiplicity of infection (MOI) on MA104 cells (African green monkey kidney epithelial cells, obtained from American Type Culture Collection (ATCC)) grown in Medium 199 (Lonza, 12–117) containing 0.1% bovine serum albumin. Viral supernatants were harvested upon cytopathic effect (CPE) (more than 60%), and cell debris was clarified by centrifugation at 500 x g for 5 min. Viral aliquots were kept at −70 °C until used.

### Virus titration by endpoint dilution assay

Viral titres were determined by an endpoint dilution assay in MA104 cells grown in 96-well plates, using 10-fold serial dilutions (from 1:10 to 5120) in septuplicate wells. CPE was assessed 2 days post-infection after cellular fixation with 4% paraformaldehyde (PFA) for 15 min and cellular staining with a solution of 0.5% crystal violet in 20% methanol for 10 min. The viral titres were calculated as tissue culture infectious doses of 50% (TCID50) per ml, in accordance with the Reed and Muench method.

### Pseudovirus neutralization assay (PNA)

The levels of neutralizing antibodies in the sera samples were determined in 96-well plates (Corning, half size) containing 16000 MA104 cells per well. Heat-inactivated serum samples were serially diluted (from 1:6 to 1:12288) in a 96-well plate in Medium 199. As controls, each plate contained wells without virus and wells with dilutions of a serum sample collected before 2019 (negative serum sample, negS). Serum dilutions were incubated with 75 TCID50 of each green florescent protein (GFP)--tagged PV for 1 h at 37 °C. At the end of the incubation, the serum–virus mixtures were added to the cells and incubated for 12 h at 37 °C. After this time, the medium was removed and the monolayers were fixed with 4% PFA for 20 min. The PFA was removed, and the cells were washed with PBS. The enhanced green fluorescent protein (eGFP) fluorescence was measured in a FLUOstar Omega Plate Reader using a basal light source, a 485/20 nm excitation filter, a 528/20 nm emission filter, and a 7x7 scan array. It was reported that in the linear range, the reduction in the GFP signal of GFP-tagged viruses is proportional to the amount of NtAbs in the serum sample [33].

The percent neutralization (PN) was calculated as follows: PN = 1 − ((PsS-bkg)/(PsnegS-bkg)), where *PsS* is the fluorescence signal obtained from cells infected with the PVs preincubated with the different dilutions of the serum sample, *PsnegS* is the signal obtained from cells infected with PVs preincubated with the different dilutions of negative serum, and *bkg* is the background fluorescence obtained from non-infected cultures. The PN results were fitted into a 4-parameter sigmoidal model using a sigmoid function (sigm_fit of MATLAB software), and the inflection points of the resulting curves were determined. The serum neutralization titre (NT_50_) was defined as the reciprocal value of the sample dilution that showed a 50% protection of reduction in GFP fluorescence. Antibodies with NT_50_titres ≥ 6 were defined as SARS-CoV-2 seropositive and neutralizing; sera with NT_50_titres < 6 were defined as negative. NtAb titres were expressed as geometric mean (interquartile range (IQR)).

### Statistical analysis

Figures and statistical analysis were conducted with MATLAB R2011a software. Wilcoxon’s rank-sum test was used to analyse changes in comparative assays. Differences between groups were analysed using the Kruskal–Wallis test with Dunn’s multiple comparison correction. In all experiments, *p* values <0.05 were considered significant.

## Results

### Study population and clinical and immunological status of the participants

The participants were classified into two groups on the day of vaccination: the infected+vaccinated (I + V) group included thirty-one people who presented IgG antibodies against the S protein before vaccination, while the naive+vaccinated (N + V) group included thirty-two participants who were seronegative to the S protein before vaccination.

Both groups included close to 70% of women and had an average age of 54 years. In the I + V group, 12.5% had type 2 diabetes (DT2), 37.5% had hypertension, 3.1% had a CVD, 3.1% had an AD, and 9.4% had a chronic RD. Moreover, 37.5% were overweight (OW), 46.8% presented obesity grades 1 to 3 (O1–O3), and 16.1% had normal weight (NW). Similar percentages of co-morbidities were observed in the N + V group (Table 1).

**Table 1.** Demographic data

		N + V group	I + V group
		N = 32	N = 31
Sex			
	Female (%)	22 (69)	21(68)
	Male (%)	10 (31)	10 (32)
Age (years)			
	Mean (IQR)	54 [51-57.5]	54.5 [52-57]
BMI classification			
	Healthy, N (%)	5 (16.1)	8 (25)
	Overweight, N (%)	12 (37.5)	11 (35.4)
	Obesity, N (%)	15 (46.8)	12 (38.6)
Co-morbidities			
	Type 2 diabetes (DT2), N (%)	6 (19.4)	4 (12.5)
	Hypertension, N (%)	8 (25.8)	12 (37.5)
	Autoimmune disease, N (%)	3 (9.7)	1 (3.1)
	Respiratory disease, N (%)	2 (6.5)	3 (9.4)
	Cardiovascular disease, N (%)	2 (6.5)	1 (3.1)
Vaccine type			
	ChAdOx1 nCoV-19, N (%)	13 (40.6)	9 (29)
	BNT162b2, N (%)	10 (31.2)	10 (32.2)
	Sputnik V, N (%)	9 (28.1)	12 (37.5)

Abbreviations: IQR, interquartile range.

The participants completed their primary vaccination regimen prior to the national peak of Delta variant infections in mid-August 2021 [34], with either Sputnik V, ChAdOx1-S, or BNT162b2 vaccines, and subsequently received a booster dose of the ChAdOx1-S vaccine (Figure 1a). Blood samples were collected between May 2021 and February 2022; the initial blood sample (PreV sample) was obtained from all participants on the day of receiving the first vaccine dose. Subsequently, most participants returned for a second and third blood draw at intervals of 10 to 41 days (30-d sample) and 93 to 146 days (120-d sample) after the second vaccine dose. The average time elapsed between the first and second doses of the vaccine was 61 days, as shown in Supplementary Table 1.

Half of the participants received a third (booster) dose of the ChAdOx1-S vaccine 168 to 207 days following the second dose. Subsequently, a post-boost (PB) serum sample was obtained from 29 participants at an interval of 23 to 34 days after the booster dose (Supplementary Table 1).

### Comparison of the anti-RBD IgG response between the N + V and I + V groups

We assessed the IgG antibody levels against the parental Wuhan strain of spike RBD protein, in the serum samples of the participants with a full primary vaccination course with either Sputnik V, ChAdOx1-S, or BNT162b2 vaccines and with a booster dose with the ChAdOx1-S vaccine using an in-house ELISA. Thirty-two (100%) of the pre-vaccination (PreV) sera from the infection-naive volunteers had undetectable (<0.12) anti-RBD IgG in the ELISA (Figure 1b,c). In contrast, 83% (25/31) of the PreV sera of the SARS-CoV-2 pre-exposed participants had anti-RBD IgG antibody levels above the positivity threshold for the ELISA (Figure 1b). Considering that the six samples that were below the positivity cut-off of the ELISA corresponded to participants who experienced a previous SARS-CoV-2 infection, all six people were classified in the I + V group.

In the N + V group, the maximal anti-RBD antibody titre after vaccination was observed 30 days after receiving the second dose of the vaccine (GM AUC = 0.93), representing a 73-fold increase compared to antibody levels in the PreV samples (GM AUC = 0.0125). At time 120d, the anti-RBD antibody levels in this group dropped 1.4 times with respect to the titre obtained 30 days after the second dose (GM AUC = 0.39) (Figure 1b).

In the I + V group, the maximal antibody response was also reached at day 30 after the second dose (GM AUC = 2.27), representing a 3.5-fold increase compared to the anti-RBD IgG observed in the PreV sera (GM AUC = 0.50). A onefold drop in the antibody level was observed (GM AUC = 1.161) 120 days after vaccination compared with time 30d (Figure 1b). The anti-RBD IgG response of I + V individuals was statistically higher than that of N + V individuals after 30d and 120d after the second dose (2.4- and fourfold higher, respectively, *p* < 0.0001).

The rate of seroconversion defined as an increase in the antibody titre ≥ 0.13 (geometric mean of the AUC values of the negative sera plus four standard deviation values) in participants of the N + V group was 97% and 81% at 30 and 120 days after vaccination, respectively. In contrast, the seropositivity rate increased up to 100% in the participants of the I + V group at both time points after vaccination (Figure 1c); these results suggest that a combination of natural infection and a full primary vaccination scheme with any of the vaccines employed elicited a greater polyclonal anti-RBD antibody response that was also of longer duration.

Half of the participants of each group (14 of 31 in the I + V group and 15 of 32 in the N + V group) opted for a third booster dose of the ChAdOx1-S vaccine. This booster dose improved the anti-RBD antibody response of participants of the N + V group, while no significant difference in the anti-RBD IgG levels was observed in the I + V group after the third vaccination. The third vaccine dose boosted the antibody levels in the N + V groups to the levels reached by people who had been infected prior to vaccination (Figure 1b). Analysis by sex revealed that there was no significant difference in the anti-RBD IgG response in males and females of both groups (data not shown). These results indicate that the booster dose is more important for people who have not been previously infected before vaccination, as opposed to those who were seropositive before vaccination.

### A natural infection followed by vaccination confers a robust neutralizing antibody response against the Alpha and Delta variants

The NtAb activity present in the participants’ sera was evaluated using replication-competent VSVs carrying the S glycoprotein of either the Wuhan, Alpha (lineage B.1.1.7), or Delta (lineage B.1.617.2) variants [32].

**Figure 2.**
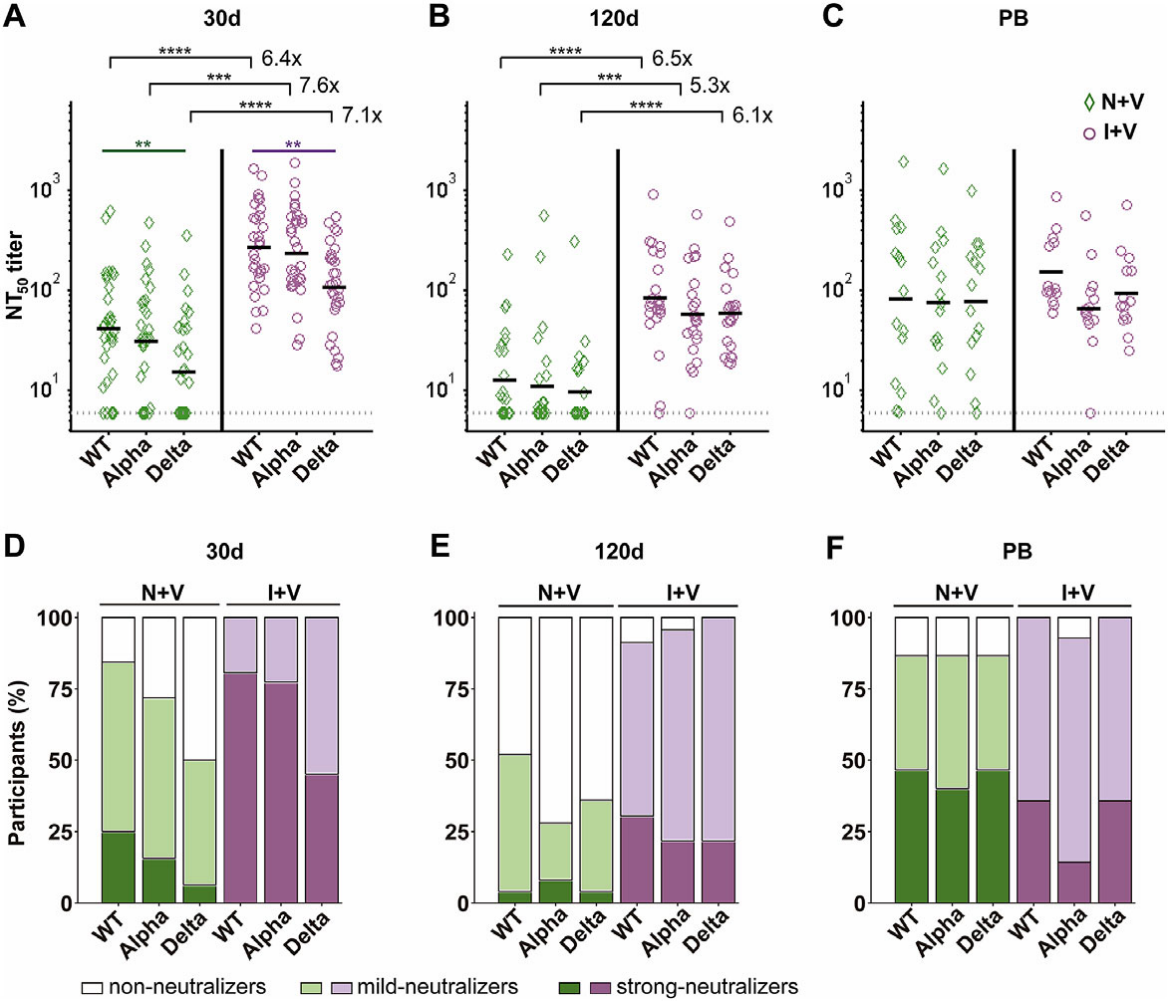
Levels of NtAb against Wuhan, Alpha, and Delta pseudoviruses in the two groups of vaccine recipients. (a)–(c) NtAb titres against pseudoviruses (PVs) bearing the SARS-CoV-2 S protein of the Wuhan, Alpha, or Delta variants, as indicated. The limit of detection of NtAb was set to the minimal dilution used (NtAb titre = 6, shown as a punctuated line). Each NT_50_ is plotted as a green diamond for the individuals in the N + V group and as a purple circle in the I + V group; lines represent the geometric mean in the N + V and I + V groups at 30d, 120d, and PB time points, respectively. The NtAb titre (NT_50_) was expressed as the maximal dilution of the sera that reduced by 50% the infectivity of the corresponding PV. A two-tailed Kruskal–Wallis test and a Dunn post hoc test were performed on log-transformed antibody data to test statistically significant differences, *p < 0.05, **p < 0.01, ***p < 0.001, ****p < 0.0001. (d)–(f) Bar plots showing the percentage of sera at 30d and 120d after full primary vaccination and 30d after the boost exhibiting non-, mild-, or strong-neutralizing activity against Wuhan-PV, Alpha-PV, and Delta-PV of the N + V and I + V groups. The cut-offs were non-neutralizing: NT50 < 6, mild neutralizer: NT50 > 6 < 120 (3rd quartile of NtAb titres against Wuhan pseudovirus at 30 days after the second dose), and strong neutralizer: NT50 > 120.

All serum samples (either N + V or I + V) collected 30 days after vaccination were less effective at neutralizing the Delta PV (Delta-PV) (1.5- to 1.8-fold reduction as compared with the Wuhan-PV, *p* < 0.01). In fact, a categorical classification of the sera based on their neutralization capacity showed that 50% of the samples in the N + V group failed to neutralize the Delta-PV. On the other hand, NtAb titres against the Alpha-PV were similar to those obtained against the Wuhan-PV, as previously reported [23, 24]; at this time (30d), 16% and 28% of the N + V volunteers showed no activity against the Wuhan-PV and Alpha-PV, respectively (Figure 2a,d and Table 2).

**Table 2.** Geometric mean of NtAb titres for the different pseudotyped variants

	30d after 2 doses	120d after 2 doses	30d after booster dose
	N + V group		
Wuhan	42.3 [18.3-121.5]	13.0 [6.0-26.4]	83.0 [17.6-380.5]
Alpha	31.4 [6.3-78.6]	11.2 [6.0-13.5]	76.6 [29.8-255.7]
Delta	15.6 [6.0-41.6]	10.0 [6.0-16.4]	79.9 [31.4.0-240.7]
	I + V group		
Wuhan	271.1 [152.4-566.1]	84.6 [60-220.2]	153.7 [95.2-306.5]
Alpha	240.1 [123.8-524.2]	58.8 [34.4-111.4]	67.8 [50.7-97.2]
Delta	110.8 [72.4-215.0]	61.0 [29.0-96.9]	94.9 [52.7-161.7]

*Note*: GM NT_50_ titres against pseudotyped variants and interquartile range (IQR) range are shown.

Interestingly, vaccination with either of the vaccines in the group of individuals who had prior exposure to SARS-CoV-2 (I + V) resulted in a significant (*p* < 0.0001) increase in the NtAb titres 30 days after completion of the full vaccination scheme of 6.4–7.6 times for the Wuhan-PV, Alpha-PV, and Delta-PV, compared with the N + V participants (Figure 2a, Table 2). At time 120d, the NtAb titres decreased in both groups, although those in the I + V group remained 5.3 to 6.5 times higher as compared to the N + V group (Figure 2b, Table 2). Hence, most serum samples of the I + V group maintained moderate to strong neutralization capacity against the Alpha-PV (96%) and Delta-PV (100%), while at time 120d, 28% and 36% of the serum samples in the N + V group showed neutralizing activity against the Alpha-PV and Delta-PV, respectively (Figure 2e).

On the other hand, the booster dose increased the NtAb titres in the N + V group 5.4, 5.8, and 7 times against Wuhan, Alpha, and Delta variants, respectively, compared with the levels observed at 120 days post-vaccination. In contrast, the increase in NtAb titres in the I + V participants was only marginal for Delta-PV and Wuhan-PV (0.6–0.8-fold) or remained the same for Alpha-PV (Figure 2c, Table 2). Of interest, the booster dose increased the neutralization response of the N + V group up to the levels reached in the I + V group (Figure 2c,f). Taken together, these results suggest that the neutralization capacity against Wuhan-PV, Alpha-PV, and Delta-PV is more potent and robust after vaccination, in individuals who had experienced a prior natural infection, and that a booster dose is more important for people who do not have hybrid immunity.

### The neutralizing antibody level induced by the ChAdOx1-S vaccine in the infection-naive group is weaker as compared to the BNT162b2 immunogen

**Figure 3.**
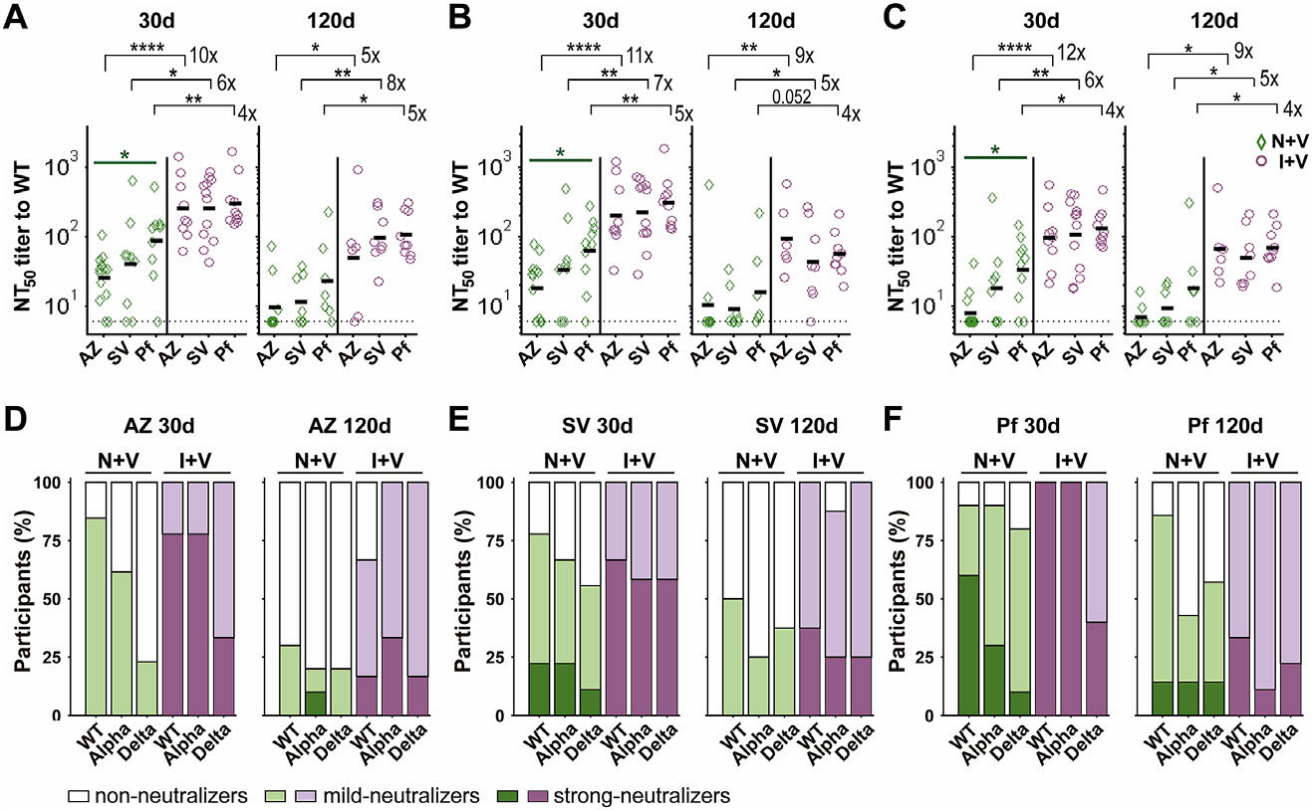
Neutralizing antibody titres against Wuhan, Alpha, and Delta pseudoviruses after vaccination with ChAdOx1-S, Sputnik V, and BNT162b2 vaccines. (a)–(c) Serum NtAb titres against the Wuhan (A), Alpha (B), or Delta (C) pseudoviruses, elicited by the ChAdOx1-S (AZ), Sputnik V (SV), or BNT162b2 (Pf) vaccines at the indicated time points. The limit of detection of NtAb was set to the minimal dilution used (NtAb titre = 6, shown as a punctuated line). Each NT50 is plotted as a green diamond for the individuals in the N + V group and as a purple circle in the I + V group; lines represent the geometric mean in the N + V and I + V groups at 30d, 120d, and PB time points, respectively. The NtAb titre (NT50) was expressed as the maximal dilution of the sera that reduced by 50% the infectivity of the corresponding PV. The number of vaccine recipients in the infection-naive N + V and I + V groups at 30d was 13 and 9 for ChAdOx1-S; 9 and 12 for Sputnik V; and 10 and 10 for the BNT162b2 vaccine, respectively. At 120d, the number of ChAdOx1-S recipients in the N + V or I + V groups was 10 and 6 for ChAdOx1-S; 8 and 8 for Sputnik V; and 7 and 9 for BNT162b2, respectively. Wilcoxon’s rank-sum test was performed to test statistical differences between N + V and I + V samples, and a two-tailed Kruskal–Wallis test and a Dunn post hoc test were used to test differences between vaccine types,****p < 0.0001,*p < 0.05, **p < 0.01, ***p < 0.001, ****p < 0.0001. (d)–(f) Percentages of sera with none-, mild-, or strong-neutralizing activity against the Wuhan-PV, Alpha-PV, or Delta-PV in the N + V and I + V groups 30 and 120 days after vaccination with AZ (D), SV (E), or Pf (F) vaccines. The cut-offs were non-neutralizing: NT50 < 6, mild neutralizer: NT50 > 6 < 120 (3rd quartile of NtAb titres against Wuhan pseudovirus at 30 days after the second dose), and strong neutralizer: NT50 > 120.

The NtAb levels induced by the three vaccines administered were compared at 30 and 120 days after the second dose in the I + V and N + V groups. In the N + V group, two doses of ChAdOx1-S induced three to four times lower NtAb levels than two doses of BNT162b2 (*p* < 0.05) at time 30d against all three PVs (Figure 3a–c). This latter vaccine induced higher NtAb responses than two doses of the Sputnik V vaccine, although the difference between BNT162b2 and Sputnik V vaccines was not statistically significant. No significant differences were found among the three vaccines after 120 days of the second dose (Figure 3a–c).

Interestingly, among the individuals with previous exposure to SARS-CoV-2, the homologous scheme with ChAdOx1-S, Sputnik V, or BNT162b2 induced similar, potent NtAb responses against the three variants analysed (Figure 3a–c). At 30 days after the second dose, pre-exposed ChAdOx1-S vaccinees showed NtAb titres 10 to 12 times higher than infection-naive ChAdOx1-S vaccines against the three PVs tested (*p* < 0.0001). Analysis at 120 days after the second dose showed a similar trend, with nine times more neutralization activity against the Delta-PV or Alpha-PV and five times more against the Wuhan-PV (*p* < 0.05).

At 30 days post-vaccination, the Sputnik V vaccinees in the I + V group showed 5.8 to 6.7 times more NtAb levels against the three PVs tested as compared to the infection-naive group. At 120 days, higher NtAb levels (five times and eight times) in the Alpha-PV or Delta-PV and Wuhan-PV were still observed in SARS-CoV-2 pre-exposed Sputnik V vaccines (*p* < 0.05). The administration of BNT162b2 to individuals in the I + V group resulted in NtAb titres four to five times higher than the Wuhan-PV, Alpha-PV, and Delta-PV at 30 days and 120 days of the second dose compared with the N + V BNT162b2 vaccinees (*p* < 0.05, except Alpha-PV at 120d).

Most of the I + V participants in the three groups of vaccinees showed detectable NtAb against the three PVs after 120 days of the second dose (Figure 3d–f **)**. In contrast, in the N + V group, only 20% of the ChAdOx1-S vaccinees, 25–38% of the Sputnik V vaccinees, and 42–56% of the BNT162b2 vaccinees retained the neutralization activity against the Alpha-PV and Delta-PV after 120 days of the second dose (Figure 3d–f). These results suggest that although the ChAdOx1-S vaccine was not as effective as the BNT162b2 vaccine in inducing NtAb against the Delta-PV in the N + V group, in those individuals primed with a natural infection the ChAdOx1-S vaccine resulted in the induction of an equivalent response as that found in the BNT162b2 vaccinees.

## Discussion

Our country was severely affected during the early circulation of SARS-CoV-2, particularly when the COVID-19 vaccines were still being developed [35]. As soon as the vaccines were available, health workers, elderly people with and without co-morbidities, and adults aged 50 to 59 years living with co-morbidities were promptly vaccinated with two doses of either BNT162b2, ChAdOx1-S, or Sputnik V (among other vaccines) from May to August 2021 [4].

A vast number of studies have analysed the immunogenicity and effectiveness of homologous schemes using the ChAdOx1-S or BNT162b2 vaccines, but there is limited information about the immunogenicity of these schemes in developing countries facing a high prevalence of co-morbidities [36]. In this study, we analysed the immunogenicity of homologous schemes using adenovirus-based (ChAdOx1-S or Sputnik V) or mRNA-based (BNT162b2) vaccines, followed by a booster dose with the ChAdOx1-S vaccine, in individuals who have had or not had prior exposure to SARS-CoV-2.

The anti-RBD IgG data showed that the seroprevalence was high and similar after 30 days of vaccination [31/32(97%) in the N + V group and 31/31 (100%) in the I + V group]. Consistent with previous reports, we observed a superior humoral response following primary immunization in the group that had a prior SARS-CoV-2 exposure [9, 37]. In these conditions, antigen-specific memory B cells are poised to quickly respond to antigens upon recall [11]. All the participants in the I + V group showed moderate or strong neutralization capacities against the Delta-PV after a two-dose vaccination scheme. In contrast, only 50% of the infection-naive participants had detectable NtAb against this virus 30 days after vaccination.

A booster dose after the full primary vaccination scheme substantially increased the NtAb response in the N + V group participants, to levels similar to those reached in the I + V group, as previously reported [9, 10, 37]. The booster dose did not increase the neutralization titre in those who had prior exposure to the virus and a full vaccination scheme. These results call into question the benefit of a third dose of vaccine in individuals with hybrid immunity and stress the need for a booster dose for those vaccinated but who had not been previously infected with the virus. Of interest, after the booster dose with the ChAdOx1-S vaccine, similar NtAb titres were obtained for the Wuhan, Alpha, and Delta variants in both groups.

Our correlation studies suggest that NtAb against the parental SARS-CoV-2 strain induced by vaccination could be inferred from the titre of IgG-binding antibodies measured in an ELISA test against the Wuhan S-RBD (data not shown). This has been previously reported [38], and it is not surprising since it is well known that the RBD domain is the main target of potent NtAb responses against SARS-CoV-2 [14].

Our results also confirm reduced NtAb levels in infection-naive individuals against the Delta variant after ChAdOx1-S vaccination [39]. At 120 days after the second dose, the percentages of non-neutralizers to the Alpha-PV and Delta-PV employed were 80% for those who received the ChAdOx1-S vaccine, 62–75% for Sputnik V, and 44–58% for BNT162b2 (Figure 3d–f). In other studies, a waning of NtAb has been observed 4 to 6 months after a full primary vaccination [40]. Interestingly, a complete vaccination scheme with the ChAdOx1-S vaccine provides a response similar to that induced by the BNT162b2 vaccine when given to individuals whose immune system was already primed by a natural infection.

One of the limitations of our study is that all the participants were middle-aged, and only the antibody immune response was characterized, while the specific immune cellular response, which is known to also mediate immunity to COVID-19, was not analysed. In addition, the reduced number of enrolled participants who were immunized with each of the three vaccines studied prevented us from having a clearer picture of the particular antibody response induced by the various vaccines in healthy people and people with different types of co-morbidities.

## Conclusions

Our results indicated that a natural exposure to SARS-CoV-2, followed by vaccination, provided superior and more stable antibody responses against the Wuhan, Alpha, and Delta variants, than the primary vaccination scheme alone. Also, it was shown that a booster dose of ChAdOx1-S in infection-naive individuals conferred similar neutralization activity against variants as that of individuals with hybrid immunity. Additional studies are required to understand the humoral immune responses of people with different co-morbidities.

## Supporting information

Garay et al. supplementary material Garay et al. supplementary material

## Data Availability

Other data supporting the results reported in this study will be available upon request by contacting the first author (EG) by email (erika.garay@ibt.unam.mx).

## Supplementary material

For supplementary material accompanying this paper visit http://doi.org/10.1017/S0950268824000219. click here to view supplementary material
