# Supplementary material for: Immune response to SARS-CoV-2 variants after immunization with different vaccines in Mexico
Source: Epidemiol Infect. 2024 Feb 5;152:e30. doi: 10.1017/S0950268824000219 (PMC10894899; doi:10.1017/S0950268824000219)
Supplement: Garay et al. supplementary material [file S0950268824000219sup001.docx]

| **Supplementary Table 1. Relevant time intervals** | | | |  |  |  |  |
| --- | --- | --- | --- | --- | --- | --- | --- |
|  | | | |  | **N+V group** |  | **I+V group** |
|  | | | |  | **Days – mean [range]** |  | **Days – mean [range]** |
| **Interval between the first and second vaccine doses** | | | |  |  |  |  |
|  |  | ChAdOx1 nCoV-19 | |  | 60.7 [59-62] |  | 61.0 [60-62] |
|  |  | BNT162b2 | |  | 39.0 [38-40] |  | 39.5 [38-40] |
|  |  | Sputnik V | |  | 84.0 [80-86] |  | 86.5 [79-91] |
|  |  | All vaccines | |  | 61.2 [38-86] |  | 62.3 [38-91] |
|  |  |  | |  |  |  |  |
| **Interval between the second vaccine dose and the third (booster) dose.** | | | |  |  |  |  |
|  | | | |  | 190.5 [168-207] |  | 192.4 [168-205] |
|  | | | |  |  |  |  |
| **Blood sample collected 30 days (30d) after the completion of the full vaccination scheme.** | | | |  |  |  |  |
|  |  | ChAdOx1 nCoV-19 | |  | 39.3 [38-41] |  | 39.0 [38-40] |
|  |  | BNT162b2 | |  | 39.0 [38-40] |  | 38.5 [38-40] |
|  |  | Sputnik V | |  | 15.8 [10-21] |  | 14.5 [10-22] |
|  |  | All vaccines | |  | 32.6 [10-41] |  | 29.4 [10-40] |
|  |  |  | |  |  |  |  |
| **Blood sample collected 120 days (120d) after the completion of the full vaccination scheme** | | | |  |  |  |  |
|  |  | ChAdOx1 nCoV-19 | |  | 123.0 [122-125] |  | 123.2 [122-124] |
|  |  | BNT162b2 | |  | 145.1 [145-146] |  | 144.6 [144-146] |
|  |  | Sputnik V | |  | 98.5 [93-104] |  | 98.4 [93-105] |
|  |  | All vaccines | |  | 121.5[93-146] |  | 122.9 [93-146] |
|  |  |  | |  |  |  |  |
| **Blood sample collected after the booster dose (PB) with ChAdOx1 nCoV-19** | | | |  |  |  |  |
|  | | | |  | 31.3 [23-34] |  | 30.4 [23-33] |
|  | | | |  |  |  |  |
| **Time between the onset of symptoms and vaccination (pre-COVID-19)** | | | |  |  |  |  |
|  | | | |  | N/A |  | 175 [18-408] |
|  | | | |  |  |  |  |
| N/A, non applicable | | |  |  |  |  |  |
